# Early developmental trajectory phenotypes for risk stratification of autism spectrum disorder in very preterm infants: a machine learning approach

**DOI:** 10.1186/s13229-025-00692-y

**Published:** 2025-12-26

**Authors:** Li-Wen Chen, Yi-Tien Li, Chi-Hsiang Chu, Chin-Chin Wu, Ching-Lin Chu, Lan-Wan Wang, Han-Yi Tsai, Chung-Hsin Chiang, Chao-Ching Huang

**Affiliations:** 1 Department of Pediatrics, National Cheng Kung University Hospital, College of Medicine, National Cheng Kung University, Tainan, Taiwan; 2 Translational Imaging Research Center, Taipei Medical University, Taipei, Taiwan; 3 Research Center for Neuroscience, Taipei Medical University, Taipei, Taiwan; 4 Ph.D. Program in Medical Neuroscience, College of Medical Science and Technology, Taipei Medical University, Taipei, Taiwan; 5 Institue of Statistics, National University of Kaohsiung, Kaohsiung, Taiwan; 6 Department of Psychology, Kaohsiung Medical University, Kaohsiung, Taiwan; 7 Department of Medical Research, Kaohsiung Medical University Hospital, Kaohsiung, Taiwan; 8 Department of Educational Psychology and Counseling, National Pingtung University, Pingtung, Taiwan; 9 Department of Pediatrics, Chi-Mei Medical Center, Tainan, Taiwan; 10 Department of Biotechnology and Food Technology, Southern Taiwan University of Science and Technology, Tainan, Taiwan; 11 School of Nursing, College of Nursing, Taipei Medical University, Taipei, Taiwan; 12 Department of Psychology, National Chengchi University, Taipei, Taiwan; 13 Research Center for Mind, Brain and Learning, National Chengchi University, Taipei, Taiwan; 14 Department of Pediatrics, College of Medicine, Taipei Medical University, Taipei, Taiwan; 15 Department of Pediatrics, Shuang Ho Hospital, Taipei Medical University, Taipei, 23561 Taiwan

**Keywords:** Preterm, Autism spectrum disorder, Neurodevelopment, Trajectory, Machine learning, Prediction

## Abstract

**Background:**

Very preterm infants are at elevated risk for autism spectrum disorder (ASD), though early identification is challenging due to overlapping neurodevelopmental disorders. While the Bayley Scales of Infant and Toddler Development (BSID) is widely used for follow-up, it remains unclear whether domain-specific developmental trajectories—such as cognition, receptive and expressive communication, and fine and gross motor function assessed by the BSID, Third Edition (BSID-III)—can support the development of a prediction model for ASD risk by preschool age in this population.

**Methods:**

This population-based multicenter cohort study included infants born < 32 weeks’ gestation in 2011–2018. Neurodevelopment was assessed at 6, 12, and 24 months using domain-specific BSID-III scaled scores. ASD diagnosis was determined at age 5 years using the Autism Diagnostic Observation Schedule and the Autism Diagnostic Interview–Revised. Infants with congenital anomalies or severe sensorimotor impairments were excluded. Developmental trajectories were analyzed using locally estimated scatterplot smoothing. Six machine learning algorithms were used to evaluate ASD prediction based on neonatal risks and longitudinal domain-specific scaled score data.

**Results:**

Of 583 very-preterm infants, 75 (12.9%) were diagnosed with ASD at preschool age. Infants later diagnosed with ASD exhibited persistently lower cognitive scores across the first two years of life (*p* < 0.05) and significantly slower development in receptive and expressive communication and fine motor skills during the second year (*p* < 0.0001 by 24 months) than infants without ASD. Gross motor trajectories did not differ significantly between groups. Integrating neurodevelopmental trajectories up to 24 months with neonatal risk factors improved prediction performance. The Support Vector Machine model yielded 71.8% accuracy (Area Under the Curve 0.69), with sensitivity of 64.2%, specificity of 72.9%, positive predictive value of 24.7%, and negative predictive value of 93.6%.

**Limitations:**

Although the model shows promise in identifying infants at low likelihood of ASD, its overall predictive performance remains modest. The model was developed in a single regional cohort, potentially limiting generalizability.

**Conclusions:**

Preterm infants later diagnosed with ASD exhibit distinct, domain-specific developmental trajectories. The model’s high negative predictive value suggests that developmental trajectory phenotypes may support early risk stratification by identifying infants at low likelihood of ASD.

**Supplementary Information:**

The online version contains supplementary material available at 10.1186/s13229-025-00692-y.

## Introduction

Autism spectrum disorder (ASD) is a neurodevelopmental condition characterized by difficulties in social communication and the presence of restricted, repetitive behaviors [1]. Signs of ASD may emerge during infancy or toddlerhood, and are typically screened between age 18 and 24 months as part of developmental surveillance for early diagnosis and intervention [2, 3]. 

Very preterm infants are at increased risk of neurodevelopmental impairments, making long-term follow-up essential for evaluating neonatal critical care quality [4, 5]. Most preterm infants undergo routine neurodevelopmental assessments at follow-up, with the Bayley Scales of Infant and Toddler Development (BSID) being the most commonly used tool at toddler age [4–7]. Children born very preterm have a significantly higher prevalence of ASD, with a 7–8% prevalence rate compared to 2–3% in the general population [8–10]. However, existing ASD screening tools often lack sufficient sensitivity and specificity for identifying the risk of ASD in preterm infants during toddler age [11–13]. Our previous study found that a low-declining cognitive trajectory from age 6 to 24 months by the BSID was associated with an increased ASD risk at age 5 years [14]. However, it remains unclear how early trajectories across multiple domains—including cognition, expressive and receptive communication, and fine and gross motor function—differ between very preterm infants who later diagnosed with ASD and those without.

Recent machine learning studies utilizing general population datasets, primarily composed of term infants, have demonstrated the predictive power of general developmental milestones and demographic/medical factors for ASD [15, 16]. Yet, the unique developmental and medical profiles remain unclear specifically for the very preterm population. Since commonly used ASD screening tools are suboptimal in this group,[11, 12] it is important to investigate whether longitudinal developmental phenotypes, as captured by the BSID, Third Edition (BSID-III)—a commonly used tool in follow-up clinics—can serve as predictors for later ASD diagnosis.

In this longitudinal cohort study of very preterm children in southern Taiwan, we conducted standardized developmental assessments using the BSID-III from 6 to 24 months of age, followed by universal autism spectrum disorder (ASD) evaluations at 5 years using the Autism Diagnostic Interview–Revised (ADI-R) and the Autism Diagnostic Observation Schedule (ADOS). The objectives of this study were twofold: (1) to characterize differences in the developmental trajectories across cognitive, language, and motor domains between children later diagnosed with ASD and those without ASD, and (2) to develop predictive machine learning models that integrate neonatal risk factors with early developmental data to forecast ASD outcomes. Through the study, we aimed to evaluate the utility of these models in stratifying ASD risk within this susceptible population.

## Methods

### Participants

This population-based multicenter cohort enrolled infants born very preterm (gestational age < 32 weeks) who were admitted to the neonatal intensive care units in a geographically defined area in southern Taiwan from January 2011 to December 2018 [14]. All infants who survived to discharge were prospectively followed for neurodevelopmental assessments at 6, 12, and 24 months of corrected age, and for ASD outcomes at 5 years of age by a cross-disciplinary team at the university hospital [9, 17]. Infants who had congenital syndromes, brain malformation, or severe sensorimotor deficits including blindness, hearing impairment, and severe cerebral palsy with gross motor function classification system score ≥ 4 (severe motor limitations with dependence on wheeled mobility for daily activities) were excluded from analysis. This study was approved by the institutional review board of National Cheng Kung University Hospital (ER-98-135). Written informed consent for data collection and neurodevelopmental follow-up was obtained from parents. This study follows the Strengthening the Reporting of Observational Studies in Epidemiology (STROBE) reporting guidelines for cohort studies.

### Demographic data and morbidities

Demographic data included gestational age, birth weight, sex, maternal educational level, and family socioeconomic status [18]. Neonatal risk factors included small for gestational age, respiratory distress syndrome requiring surfactant treatment, durations of invasive mechanical ventilation and supplemental oxygen therapy, hemodynamically-significant patent ductus arteriosus requiring surgical intervention, necrotizing enterocolitis ≥ stage II confirmed with clinical and radiological evidences, [19] sepsis documented by blood culture reports, high-grade intraventricular hemorrhage with ventriculomegaly or intraparenchymal lesion, and periventricular leukomalacia suggesting cystic white matter injuries. Diagnoses of bronchopulmonary dysplasia and severe retinopathy of prematurity were made at 36 weeks’ postmenstrual age, [20, 21] and the total length of hospital stay and extrauterine growth in weight and head circumference were recorded at discharge [22]. 

### Neurodevelopmental assessments at corrected ages 6, 12, and 24 months

Infants were assessed using the BSID-III, administered by child psychologists and examined by pediatric neurologists for neurodevelopmental disorders. The BSID-III evaluates five domains: cognition, receptive communication, expressive communication, fine motor function, and gross motor function. Raw scores from each domain were converted to age-appropriate scaled scores, with a mean of 10 and a standard deviation of 3 [23].

### ASD outcome determination at age 5 years

ASD diagnoses were confirmed by a multidisciplinary team of pediatric neurologists, child psychologists, and child psychiatrists. Diagnoses were based on evaluations using the Diagnostic and Statistical Manual of Mental Disorders, Fifth Edition (DSM-5), the Autism Diagnostic Interview–Revised (ADI-R), and the Autism Diagnostic Observation Schedule (ADOS), First Edition [9, 14, 24–26]. While ADOS Module 3 was typically administered in this cohort, Modules 1 and 2 were adopted for children with differing language abilities, with ASD diagnoses determined based on the criteria corresponding to each module. The final diagnosis was determined at the follow-up clinic through a team-based discussion that integrated the ADI-R and ADOS scores with clinical judgment. Intelligence outcome was assessed using the Wechsler Preschool and Primary Scale of Intelligence, Revised (WPPSI-R) or the Fourth Edition (WPPSI-IV) [27]. 

### Statistical analyses

Differences in demographics, neonatal morbidities, and neurodevelopmental scores between children with and without ASD were examined using the Mann–Whitney U test or Fisher’s exact test, as appropriate. Developmental trajectories based on BSID-III scaled scores for cognition, receptive and expressive communication, fine motor, and gross motor functions across corrected ages 6, 12, and 24 months were analyzed using locally estimated scatterplot smoothing (LOESS).

### Feature selection

Neonatal risk factors were selected based on differences between the ASD and no-ASD groups with *p* < 0.1, and on critical ASD-related risk factors reported in the literature [14, 28]. Scaled scores from the BSID-III at corrected ages 6, 12, and 24 months—reflecting developmental trajectories—were then sequentially incorporated into the models. Univariate logistic regression was first used to assess the association between each predictor and ASD outcome. Predictors with *p* < 0.05, adjusted with false discovery rate (FDR), were retained for further multivariable analysis. The absolute value of the t-statistic was used to quantify the importance of each feature, with *p* < 0.05 considered statistically significant. Subsequently, the least absolute shrinkage and selection operator (LASSO) logistic regression to reduce multicollinearity and to identify the most informative features was applied for feature selection. LASSO was implemented using the MATLAB Statistics and Machine Learning Toolbox, with the penalty parameter λ chosen by internal cross-validation. This method applies an L1 penalty to the regression coefficients, shrinking less informative coefficients toward zero and effectively excluding those features from the model.

For both logistic regression and LASSO, class weights were applied to address class imbalance. These weights were calculated based on the ratio of ASD to non-ASD cases, allowing the model to place greater emphasis on correctly classifying the minority class, i.e., ASD.

### Machine learning classifiers

For each set of selected features, six machine learning algorithms were trained using the MATLAB Classification Learner app (R2020a): Decision Trees, Naïve Bayes, Support Vector Machines, K-Nearest Neighbors, Discriminant Analysis, and Ensemble Learning (Supplemental Methods 1).

All hyperparameters for each algorithm were optimized using the Classification Learner app’s built-in grid search procedure, selecting the best-performing parameter set via internal cross-validation and iterating through multiple parameter combinations to identify the best-performing configuration. Misclassification costs were adjusted according to the ASD to no-ASD sample ratio (1:6.8), to increase sensitivity for the minority ASD class.

### Model evaluation

Model performance was evaluated using leave-one-out cross-validation, which maximizes data utilization given the modest number of ASD cases and provides nearly unbiased estimates of prediction error. To address class imbalance, misclassification costs were incorporated during model training. The cost function was adjusted to assign a higher penalty to misclassifications of the minority class (the ASD group), thereby improving sensitivity. The cost matrix was defined based on the ratio of ASD to non-ASD cases in the dataset.

Model performance was compared across four feature sets: Model 1: neonatal risk factors only; Model 2: neonatal risk factors plus 6-month BSID-III scaled scores; Model 3: neonatal risk factors plus 6- and 12-month scaled scores; and Model 4: neonatal risk factors plus 6-, 12-, and 24-month scaled scores. The area under the receiver operating characteristic curve (ROC-AUC) was plotted for each model. The optimal classification threshold was determined by maximizing Youden’s Index, defined as sensitivity plus specificity minus one, which identifies the cut-off that best balances true positive and true negative rates. The sensitivity and specificity at this threshold were reported for each model.

The statistics were performed using R software, version 4.4.1. Machine learning models were developed using MATLAB (version R2024a) [29, 30]. These models were trained using the MATLAB Classification Learner app, which facilitates the training and evaluation of classification models. The level of *p* < 0.05 was considered statistically significant for all analyses.

## Results

Of the 914 infants born very preterm and admitted to the neonatal intensive care units, 785 survived to discharge. Seven infants died during follow-up, and 625 (80%) completed longitudinal developmental assessments through 5 years of age (Supplemental Fig. 1). Children lost to follow-up (*n* = 153) had significantly lower family socioeconomic status and maternal educational level, and were less likely to have bronchopulmonary dysplasia and had higher z-scores for head circumference at discharge compared to those who completed follow-up (Supplemental Table 1). After excluding 42 infants with congenital disorders, post-discharge brain injury, or significant neurosensory deficits that could interfere with ASD assessment, 583 children were included in the final analysis.

### Differences in demographics, adverse neonatal exposures, and 5-year behavioral profiles between the ASD and no-ASD groups

Among the 583 children, 75 (12.9%) were diagnosed with ASD based on the ADI-R and ADOS assessments at age 5 years. Although ADOS Module 3 was generally administered to preschool-aged children, 7 of the 583 children were evaluated with Module 1 and 43 with Module 2 according to their language abilities. The ASD group was significantly more likely to be male (52 of 75, 69%) compared to the no-ASD group (247 of 508, 49%) (*p* < 0.001). Otherwise, the two groups were comparable in gestational age, birth weight, family socioeconomic status, and the proportions of neonatal risks and morbidities at discharge (Table 1). The ASD group had significantly higher scores in domains of reciprocal social interaction, communication, and restricted, repetitive, and stereotyped behaviors compared to the no-ASD group (all *p* < 0.0001) by ADI-R and ADOS (Table 1). The ASD group also had a lower median full-scale intelligence quotient than the no-ASD group (91 vs. 94, *p* = 0.03) by WPPSI.

**Table 1:** Differences in demographics, neonatal risks and morbidities, and outcomes at age 5 years between very preterm children with and without autism spectrum disorder (ASD)

	ASD, *n* = 75	No-ASD, *n* = 508	*P*-value
**Demographics**			
Gestational age, weeks, median (IQR)	28 (26–29)	28 (27–30)	0.06
Birth weight, grams, median (IQR)	1086 (780–1318)	1090 (889–1300)	0.3
Sex, male, n (%)	52 (69)	247 (49)	0.0008
Maternal educational level below university, n (%)	42 (56)	248 (49)	0.3
Low family socioeconomic status, n (%)	22 (29)	141 (28)	0.8
**Neonatal risks/morbidities**			
Small for gestational age, n (%)	9 (12)	32 (6)	0.09
RDS requiring surfactant, n (%)	30 (40)	196 (39)	0.8
IMV duration, days, median (IQR)	1 (0–9)	1 (0–6)	0.8
Supplemental O2 duration, days, median (IQR)	48 (29–85)	43 (27–67)	0.1
PDA requiring surgical intervention, n (%)	7 (9)	55 (11)	0.8
NEC ≥ stage II, n (%)	7 (9)	42 (8)	0.8
Sepsis, n (%)	14 (19)	69 (14)	0.3
High-grade IVH or PVL, n (%)	10 (13)	47 (9)	0.3
**At discharge/Postmenstrual age 36 weeks**			
Severe ROP, n (%)	10 (13)	64 (13)	0.9
BPD, n (%)	23 (31)	151 (30)	0.9
Hospital stay, days, median (IQR)	65 (50–98)	59 (47–81)	0.09
Body weight z-score, median (IQR)	-1.2 (-2.2 – -0.7)	-1.4 (-2.1 – -0.8)	0.4
Head circumference z-score, median (IQR)	-1.2 (-2.0 – -0.5)	-1.3 (-2.0 – -0.6)	0.6
**At age 5 years**			
**Autistic behaviors**			
ADI-R Domain A: Qualitative abnormalities in reciprocal social interaction, median (IQR)	10 (6–17)	1 (0–4)	< 0.0001
ADI-R Domain B: Qualitative abnormalities in communication, median (IQR)	9 (5–13)	0 (0–3)	< 0.0001
ADI-R Domain C: Restricted, repetitive, and stereotyped patterns of behavior, median (IQR)	3 (0–5)	0 (0–1)	< 0.0001
ADOS communication, median (IQR)	3 (2–5)	1 (0–2)	< 0.0001
ADOS social interaction, median (IQR)	7 (4–9)	1 (1–2)	< 0.0001
ADOS imagination/creativity, median (IQR)	1 (1–2)	0 (0–1)	< 0.0001
ADOS stereotyped behavior, median (IQR)	1 (0–2)	0 (0–1)	< 0.0001
**Intellectual function by WPPSI**			
Full-scale intelligence quotient (FSIQ), median (IQR)	91 (76–100)	94 (85–101)	0.03

By Mann-Whitney test or Fisher’s exact test. ADI-R: Autism Diagnostic Interview-Revised; ADOS: Autism Diagnostic Observation Schedule; ASD: autism spectrum disorder; BPD: bronchopulmonary dysplasia; IMV: invasive mechanical ventilation; IQR: interquartile range; IVH: intraventricular hemorrhage; NEC: necrotizing enterocolitis; PDA: patent ductus arteriosus; PVL: periventricular leukomalacia; RDS: respiratory distress syndrome; ROP: retinopathy of prematurity; WPPSI: Wechsler Preschool and Primary Scale of Intelligence

### Distinct trajectories of early-life development in infants who later diagnosed with ASD

**Fig. 1:**
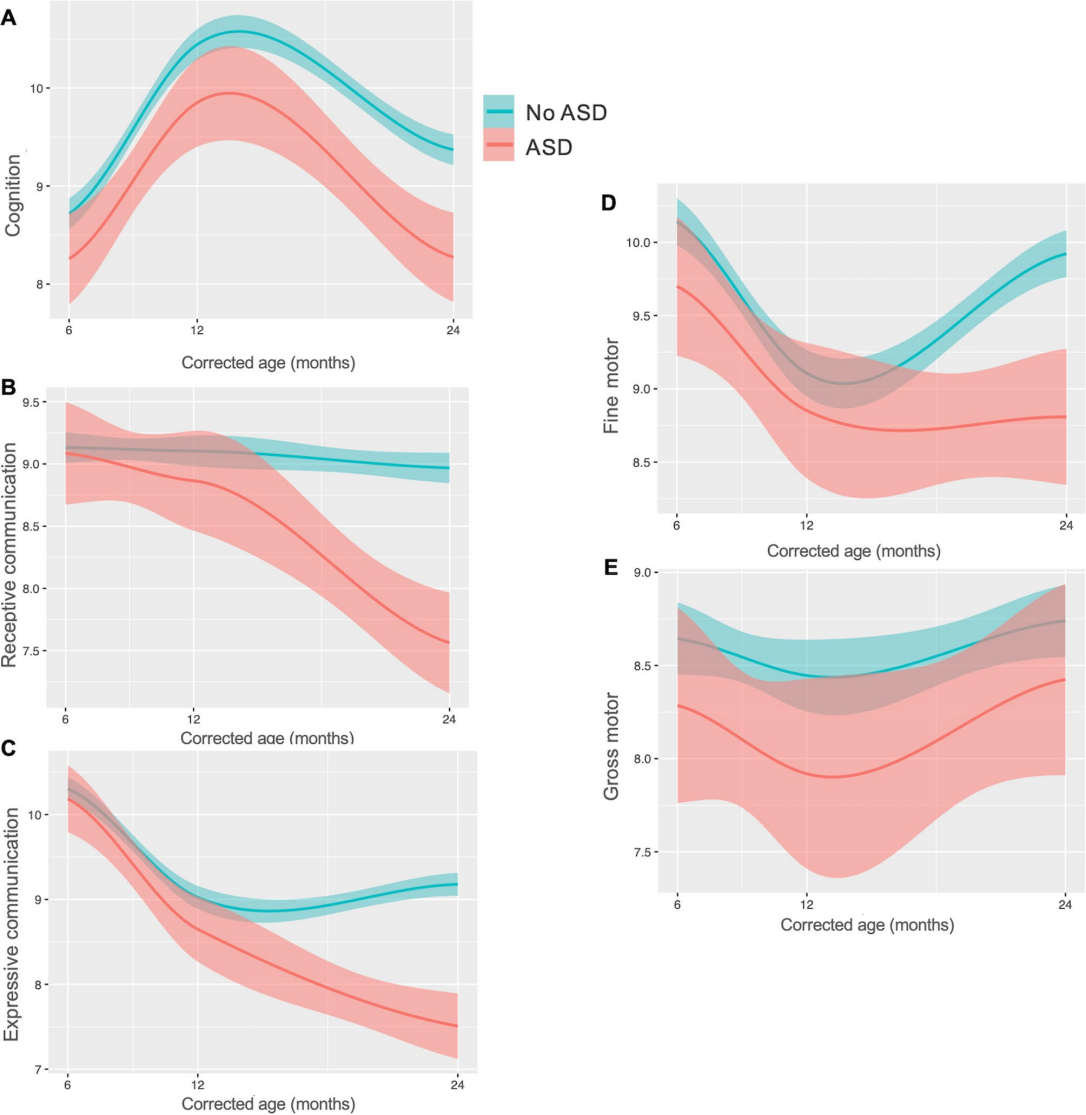
Early-life developmental trajectories of cognition (**A**), receptive (**B**) and expressive (**C**) communication, and fine (**D**) and gross (**E**) motor scaled scores from corrected age 6 to 24 months between children with and without autism spectrum disorder at 5 years. Locally estimated scatterplot smoothing (LOESS) with 95% confidence interval

First, we examined whether the ASD group exhibited distinct early developmental trajectories compared to the no-ASD group. From 6 to 24 months of age, the ASD group consistently demonstrated lower cognitive performance than the no-ASD group (*p* = 0.03 at 6 months, *p* = 0.05 at 12 months, *p* < 0.0001 at 24 months; Table 2, Supplemental Fig. 2, and Fig. 1A). In contrast, no significant differences were observed in gross motor function between the two groups (Fig. 1E). Although receptive communication, expressive communication, and fine motor skills were similar between groups from 6 to 12 months, the developmental trajectories in these domains began to diverge markedly from 12 to 24 months, with the ASD group showing significantly declining trajectory indicating slower development of skills (all *p* < 0.0001; Fig. 1B–D).

**Table 2:** Differences in the developmental composite scores and the scaled scores at corrected age 6, 12, and 24 months between children with and without ASD diagnosed at age 5 years

	6 months			12 months			24 months		
	No ASD	ASD	*p*-value	No ASD	ASD	*p*-value	No ASD	ASD	*p*-value
**Composite score, median (IQR)**									
Cognition	95 (90–100)	90 (85–95)	0.03	100 (100–110)	100 (95–105)	0.03	95 (90–105)	90 (85–100)	< 0.0001
Language	97 (97–103)	97 (94–103)	0.6	94 (91–97)	94 (91–95)	0.01	94 (89–103)	89 (77–94)	< 0.0001
Motor	97 (88–107)	96 (88–103)	0.2	94 (91–97)	91 (85–97)	0.1	97 (91–103)	94 (85–97)	0.0006
**Scaled score**,** median (IQR)**									
Cognition	9 (8–10)	8 (7–9)	0.03	10 (10–12)	10 (9–11)	0.05	9 (8–11)	8 (7–10)	< 0.0001
Receptive communication	9 (9–10)	9 (9–10)	0.8	9 (9–9)	9 (9–9)	0.07	9 (8–10)	8 (6–9)	< 0.0001
Expressive communication	10 (10–11)	10 (10–11)	0.1	9 (8–10)	9 (8–10)	0.04	10 (8–11)	8 (6–9)	< 0.0001
Fine motor	11 (8–12)	10 (8–11)	0.2	9 (9–10)	9 (8–10)	0.1	10 (9–11)	9 (8–10)	< 0.0001
Gross motor	9 (7–10)	8 (7–10)	0.2	9 (8–9)	8 (7–9)	0.2	9 (8–10)	9 (8–9)	0.1

Scaled scores in median (interquartile range). Compared by Mann-Whitney tests. The composite scores and the scaled scores were by the Bayley Scale of Infant and Toddler Development, Third Edition (BSID-III)

### Prediction of ASD diagnoses at age 5 years using neonatal risks and developmental phenotypes at 6, 12, and 24 months

Next, we evaluated whether neonatal risk factors and developmental phenotypes at 6, 12, and 24 months could predict preschool-age ASD outcomes using various machine learning models. Predictors identified through LASSO regression were incorporated to optimize prediction accuracy. First, based on Model 1: neonatal risk factors only, predictors selected by the LASSO method included gestational age, sex, small-for-gestational-age status, and maternal educational level. Among all models tested, the K-Nearest Neighbors classifier demonstrated the best performance when using neonatal risk factors alone, achieving an accuracy of 60.2% and an ROC-AUC of 0.57 (Table 3).

**Table 3:** Model performances for prediction of autism spectrum disorder (ASD) at age 5 years using neonatal risks and developmental phenotypes at corrected-age 6, 12, and 24 months by the Bayley scale of infant and toddler development, third edition (BSID-III)

Feature factors	Model	ROC-AUC	Accuracy (%)	Sensitivity (%)	Specificity (%)
Model 1: Neonatal risks*	Decision Tree	0.47	53.1	67.2	51.1
	Support Vector Machine	0.61	53.1	67.2	51.1
	Naïve Bayes Classifier	0.57	49.7	31.3	52.2
	K-Nearest Neighbor Classifier	0.57	60.2	53.7	61.1
	Discriminant Analysis	0.59	57.3	58.2	57.1
	Ensemble Classifier	0.48	53.1	67.2	51.1
Model 4. Neonatal risks plus the BSID-III scaled scores at 6, 12, and 24 months**	Decision Tree	0.60	64.4	55.2	65.6
	Support Vector Machine	0.69	71.8	64.2	72.9
	Naïve Bayes Classifier	0.65	66.0	59.7	66.9
	K-Nearest Neighbor Classifier	0.71	64.2	71.6	63.1
	Discriminant Analysis	0.69	68.5	61.2	69.6
	Ensemble Classifier	0.61	67.3	61.2	68.1

*Selected features: gestational age, sex, small for gestational age, and maternal educational level. **Selected features: gestational age, sex, small for gestational age, 6-month scaled scores of cognition and receptive communication, 12-month scaled score of receptive communication, and 24-month scaled scores of receptive communication, expressive communication, fine motor, and gross motor function. ROC-AUC: the area under the receiver operating characteristic curve

**Fig. 2:**
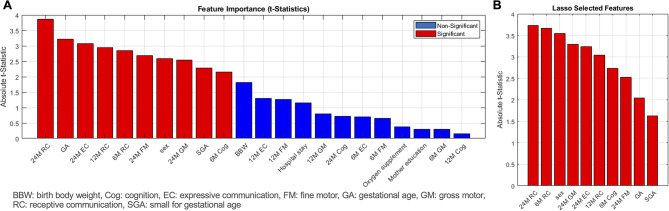
Significant predictors for ASD by logistic regression analysis (**A**) and the LASSO method (**B**). Among the variables, 10 were selected as significant predictors by logistic regression analysis, which were further confirmed by the LASSO method

**Fig. 3:**
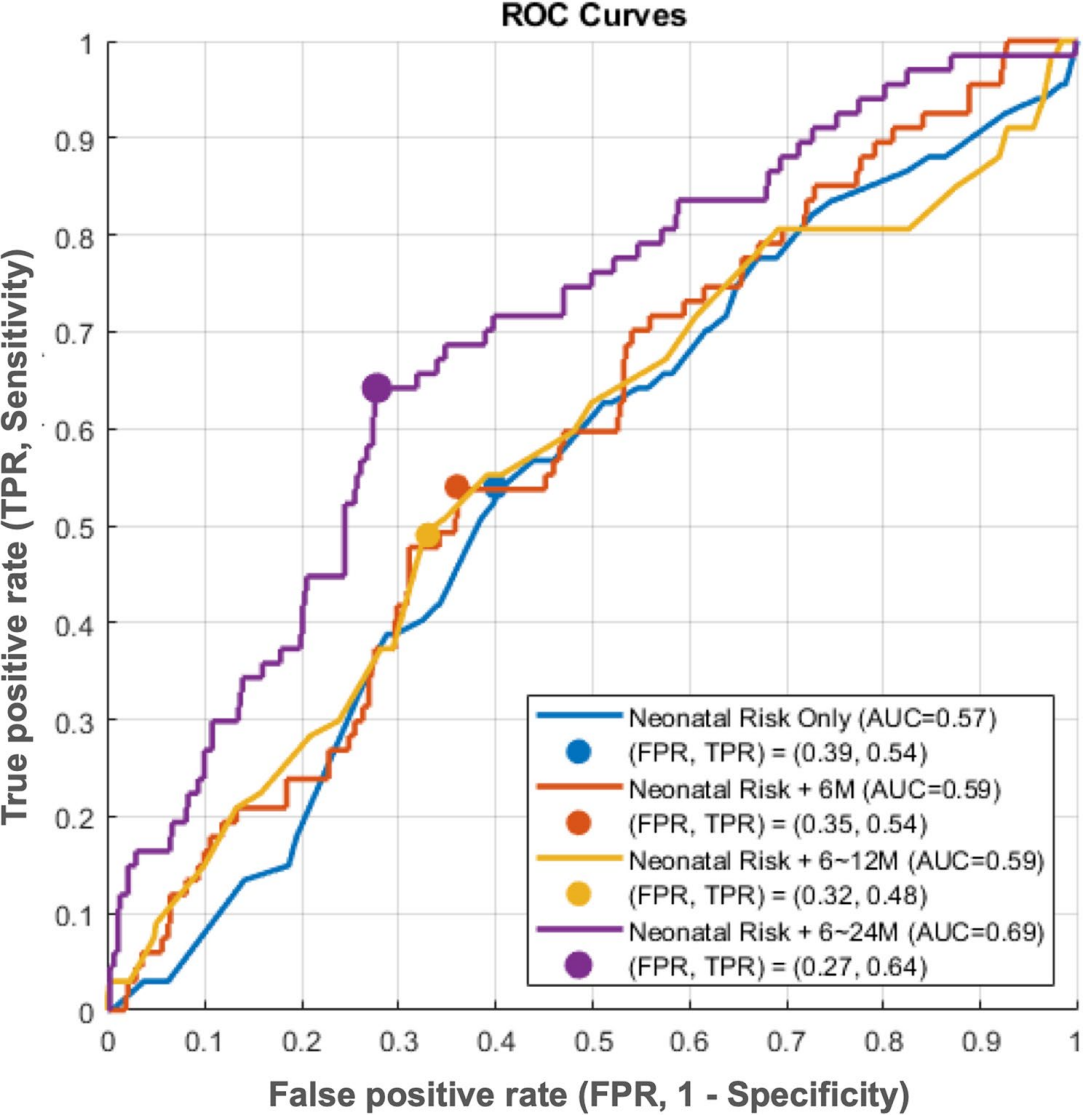
Comparisons of the best-performing ROC curves with optimal thresholds using 4 different sets of features. Blue: neonatal risk factors only; Orange: neonatal risk factors and 6-month BSID-III scaled scores; Yellow: neonatal risk factors and 6- and 12-month BSID-III scaled scores; and Purple: neonatal risk factors and 6-, 12-, and 24-month BSID-III scaled scores. Adding the 24-month developmental scores (purple) significantly improved the performance of prediction model

**Fig. 4:**
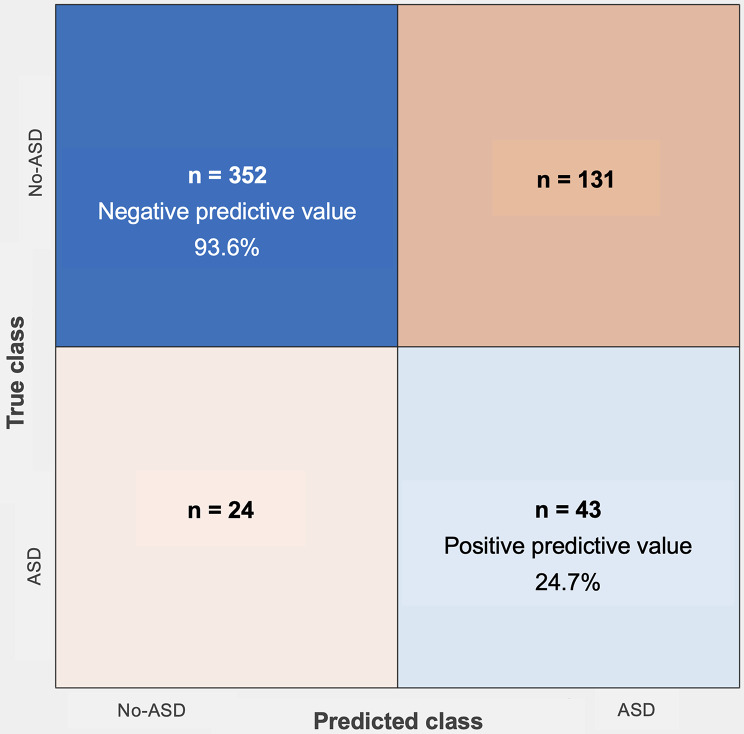
Confusion matrix to summarize the best classification model using neonatal risks and developmental scores from 6, 12, to 24 months by the Support Vector Machine classifier

Next, we examined whether sequential incorporation of scaled scores for cognition, receptive communication, expressive communication, fine motor, and gross motor function at 6, 12, and 24 months could enhance the predictive power for ASD. Incorporating developmental phenotypes at 6 and 12 months (Models 2 and 3) resulted in only marginal improvements in model performance (Supplemental Table 2). Based on the dataset of Model 4: neonatal risk factors plus 6-, 12-, and 24-month BSID-III scaled scores, 10 predictor features were selected by the LASSO method, including gestational age, sex, small-for-gestational-age status, scaled scores for cognition and receptive communication at 6 months, receptive communication at 12 months, and receptive communication, expressive communication, fine motor, and gross motor function at 24 months (Fig. 2A and B). The prediction accuracy and ROC-AUC increased substantially using these 10 predictors, compared with the performances based on neonatal risks and developmental data prior to 24 months of age (Fig. 3). The Support Vector Machine classifier achieved the best overall performance, with an accuracy of 71.8% and an ROC-AUC of 0.69 (sensitivity: 64.2%; specificity: 72.9%; positive predictive value: 24.7%; negative predictive value: 93.6%) (Table 3; Fig. 4).

## Discussion

Early identification of very preterm infants at high risk for later ASD diagnosis remains a significant challenge for early intervention. The BSID-III is a commonly applied tool for developmental evaluations in every very preterm infant world-wide. In this longitudinal very preterm cohort, we found that infants who later diagnosed as ASD by preschool age exhibited distinct developmental trajectories before the age of 2 years. Specifically, the ASD group showed significantly lower cognitive performance from 6 to 24 months, as well as reduced receptive and expressive communication and fine motor skills between 12 and 24 months, compared to peers without ASD. Moreover, developmental domain patterns—particularly at 24 months—substantially enhanced the predictive accuracy of ASD diagnosis using machine learning algorithms. Given the model’s high negative predictive value (93.6%) and low positive predictive value (24.7%), it may be most useful as a risk stratification tool to identify preterm infants at low likelihood of ASD, thereby reducing unnecessary referrals and optimizing resource use.

### Characteristic developmental trajectory phenotypes in preterm infants who later diagnosed with ASD

Early-life developmental cascades may provide insights into the emergence of developmental differences between children with and without ASD. Children with ASD often exhibit characteristic deviations in cognition, communication, and motor function compared to those with typical development [31–33]. However, most of these studies that primarily focused on term-born infants have examined longitudinal developmental patterns associated with ASD by comparing children with ASD to their siblings without ASD [34–37]. The limited availability of direct data on the early developmental trajectory characteristics in very preterm infants who are later diagnosed with ASD makes early detection more challenging [38]. Our study is the first to demonstrate the distinct differences in early developmental trajectories between children born very preterm who developed ASD at preschool age and their peers without ASD. The discrepant cognitive and communication developmental trajectories are in line with previous studies in preterm and term babies [14, 34, 36]. However, as motor delay frequently precedes ASD diagnosis that may help early identification in general poulation, [39] our study specified that in very preterm infants, fine motor ability may provide more clues for ASD than gross motor function. Because the evaluation of fine motor function in the BSID-III often requires imitation, the lower fine motor scores observed in preterm infants with ASD may partly reflect difficulties in interaction between the child and the examiner.

### Predictive models for ASD in general population-based studies

Several predictive models using developmental surveillance data have been developed for ASD diagnosis in the general population, and might surpass ASD-specific screening tools in predictive accuracy [15, 16]. However, these models either excluded very preterm infants or included only a small number of them. For instance, a nationwide developmental assessment study in Israel, which analyzed data from 1.2 million children while excluding those born before 34 weeks’ gestation, produced a prediction model with 45% sensitivity and 95% specificity, outperforming the Modified Checklist for Autism in Toddlers (M-CHAT) screening questionnaire [15]. Another model based on over 30,000 subjects and 28 questions on medical and background history identified developmental milestones and eating behavior as key predictors, demonstrating accuracy and generalizability for ASD prediction [16]. These studies, primarily focused on term-birth infants, align with our findings that general developmental patterns can provide critical insights for early ASD detection. Our model is the first to apply machine learning algorithms specifically for ASD prediction in the very preterm population. Using 10 variables, i.e. 3 neonatal risks (gestational age, sex, and small-for-gestational-age status) and 7 longitudinal developmental scores until age 24 months by the BSID-III, our model achieved a sensitivity of 64.2% and a specificity of 72.9%.

### Comparisons with screening tools for ASD detection in preterm population

Screening for ASD in preterm infants is even more challenging than in the general population due to the high prevalence of various neurodevelopmental disorders in this population [11, 40, 41]. For instance, studies using the 23-question M-CHAT have reported a sensitivity of 52% and a specificity of 84% for ASD in extremely preterm infants [11]. Recently, the updated 20-question M-CHAT, Revised With Follow-Up, demonstrated improved sensitivity and specificity [42]. To minimize the impact of sensorimotor impairments on ASD diagnosis, our study excluded infants with cerebral palsy and hearing or visual impairments from the analysis. Compared to previous reports on M-CHAT study in preterm population, our prediction model has shown comparable sensitivity, making it a viable option for ASD risk stratification in clinical settings.

### The machine learning predictive model for ASD in very preterm population

The predictive performance of the SVM model in this very preterm cohort may be promising, given the complexity of early ASD identification in the preterm population. While the model’s moderate sensitivity and specificity offer a balanced classification, the relatively low positive predictive value indicates limited reliability in identifying true ASD cases. In contrast, the high negative predictive value underscores its potential utility in identifying infants at low risk of ASD, which may reduce the anxiety of parents while avoiding overuse of medical resources.

In clinical practice, such a model could be integrated as a triage tool to support decision-making on surveillance intensity rather than as a standalone diagnostic pathway. It may serve to complement existing screeners, such as the M-CHAT, by refining follow-up prioritization, especially in contexts where traditional tools underperform in preterm populations [11–13, 40, 41, 43]. Furthermore, the model threshold could be calibrated to emphasize sensitivity or specificity depending on clinical objectives and resource constraints, enhancing adaptability across diverse care settings. The model may also assist clinicians in identifying subthreshold yet persistent developmental delays in very preterm infants who do not meet criteria by early screening tools for ASD, supporting more informed decisions about ongoing surveillance and early intervention in follow-up care.

### Identifying risk factors, temporal changes in developmental domains and prediction models for ASD in preterm infants

Large cohort studies have identified risk factors for ASD in preterm infants; however, these studies often lacked the analytical power to account for the heterogeneity of neonatal risk factors and the variability in early developmental patterns [43–45]. Our study addressed these limitations by selecting clinically significant variables specific to very preterm infants and applying the LASSO method for an unbiased identification of key predictors. In addition to the demographics and neonatal risks, adding the developmental domain data of receptive communication, expressive communication, fine motor, and gross motor function at 24 months greatly improved the prediction performance. By comparing multiple machine learning models with different sets of variables and incorporating temporal changes in developmental trajectories, we were able to validate the predictive accuracy and to determine the optimal age for ASD prediction in this high-risk population.

### The high ASD prevalence rate and better cognitive scores

The 12.9% rate of ASD diagnoses observed in the current study is higher than the generally reported prevalence of 7% in the literature [8]. However, more recent studies have reported the ASD prevalence ranging from 12.7% to 20.8%. [46–48] The rising trend in ASD diagnoses over time may reflect advances in perinatal and neonatal care that have improved the survival of critically-ill infants, as well as greater awareness of ASD susceptibility among preterm populations which may facilitate earlier and more accurate detection.

The cognitive scores in our study appeared higher than those reported in other cohorts of preterm infants with ASD [11, 49, 50]. However, as our cohort comprised very preterm infants born in the recent decade and excluded those with congenital malformations or severe sensorimotor deficits, the comparatively better scores may reflect differences in study populations, particularly when compared with cohorts enrolling extremely preterm infants. In addition, our study implemented universal ASD diagnostic assessments, in contrast to the approaches by others that apply a gated screening threshold to determine eligibility for diagnostic evaluation [45, 50]. Consequently, children with ASD identified in our cohort may exhibit higher levels of functioning compared with those reported in other studies, where diagnostic evaluation was limited to children who did not pass the screening.

### Limitations and strengths

Our study had limitations. First, although the Support Vector Machine model demonstrated a high negative predictive value, indicating strong potential for identifying preterm infants at low likelihood of ASD, its overall predictive performance remains modest. The model’s moderate sensitivity and low positive predictive value suggest limited accuracy in identifying preterm infants who will later be diagnosed with ASD. These challenges may reflect the heterogeneity of ASD presentations and the complexity of early developmental trajectories in preterm populations. Second, we implemented a universal ASD diagnostic assessment at age 5 years in this research cohort—later than the commonly recommended window—based on prior evidence that screening tools and ADOS assessments in preterm infants aged 2–4 years often have limited accuracy and low detection rates [12]. However, in clinical practice, children who exhibited concerns in neurodevelopment including the socioemotional domain at any follow-up visit (6, 12, or 24 months) were referred to early intervention programs. Additionally, the model was trained and tested within a single regional cohort, which may limit generalizability. Larger preterm cohorts are needed to elucidate the interaction between intellectual function and ASD diagnosis,[45, 50] both of which are key outcomes in the long-term follow-up of preterm infants. External validation in diverse populations and incorporation of additional risk markers—such as neuroimaging or genetic data—may enhance predictive precision in future studies.

Despite these limitations, our study also had notable strengths. First, ASD diagnostic assessments using the ADI-R and ADOS were routinely conducted for all children born very preterm at age 5 years, ensuring diagnostic consistency. Second, use of the BSID-III provides a feasible approach facilitating early ASD risk detection in most preterm follow-up clinics. This study, conducted within Taiwan’s structured neonatal follow-up system with standardized BSID-III assessments, may be generalizable to other health systems with similar infrastructure and to very preterm populations without significant congenital malformation or sensorimotor deficits [17]. Applications using the newer tools such as the BSID, Fourth Edition, or with limited data points (e.g., only at 18–24 months), requires further validation. Based on developmental data up to 24 months, this prediction model that stratified ASD risk could serve as a decision support tool at follow-up visits to guide for diagnostic evaluations such as the ADOS. Rather than replacing existing screeners for ASD, with further refinement, the prediction model may evolve into a numeric risk score based on clinical context to optimize resource allocation.

## Conclusion

Very preterm-birth children with ASD diagnosed at preschool age is preceded by distinct developmental trajectories in cognition, communication, and fine motor function during the first two years of life. A machine learning model incorporating different developmental domain trajectories across the first 24 months may assist clinicians in identifying children at low risk for ASD.

## Supplementary Information

Below is the link to the electronic supplementary material.

Supplementary Material 1

## Data Availability

The datasets generated and analyzed during the current study are not publicly available due to consent limitations, but are available from the corresponding author on reasonable request.

## Abbreviations

ADI-R: Autism Diagnostic Interview-Revised
ADOS: Autism Diagnostic Observation Schedule
ASD: Autism spectrum disorder
BSID-III: Bayley Scales of Infant and Toddler Development, Third Edition
LASSO: Least absolute shrinkage and selection operator
LOESS: Locally estimated scatterplot smoothing
M-CHAT: Modified Checklist for Autism in Toddlers
ROC-AUC: Area under the receiver operating characteristic curves
