# Supplementary material for: Early developmental trajectory phenotypes for risk stratification of autism spectrum disorder in very preterm infants: a machine learning approach
Source: Mol Autism. 2025 Dec 26;17:3. doi: 10.1186/s13229-025-00692-y (PMC12805792; doi:10.1186/s13229-025-00692-y)
Supplement: Supplementary file 1 — Supplementary Material 1 [file 13229_2025_692_MOESM1_ESM.docx]

**Supplemental Material**

**Page 2 –** Supplemental Methods. Machine Learning Classifiers

**Page 3 –** Supplemental Figure 1. Patient enrollment

**Page 4 –** Supplemental Table 1. Demographics, neonatal risks and morbidities between children with follow-up and children lost to follow-up.

**Page 6** – Supplemental Figure 2. Box plots of BSID-III scaled scores at 6, 12, and 24 months in the no-ASD and ASD groups.

**Page 7** – Supplemental Table 2. Model performances for prediction of autism spectrum disorder (ASD) using neonatal risks and developmental phenotypes at 6 and 12 months by the Bayley Scale of Infant and Toddler Development, Third Edition (BSID-III).

**Supplemental Methods.** Machine Learning Classifiers

Six supervised learning classifiers were trained using the MATLAB Classification Learner app (R2020a):

- **Decision Tree**: split criterion - Gini’s diversity index, automatic maximum depth determined by cross-validation.
- **Naïve Bayes**: Gaussian kernel density estimation.
- **Support Vector Machine (SVM)**: radial basis function kernel, box constraint and kernel scale automatically optimized by grid search in the app.
- **K-Nearest Neighbors (KNN)**: Euclidean distance, k automatically selected from 1–30 by cross-validation.
- **Discriminant Analysis**: linear and quadratic discriminant functions, with automatic model selection.
- **Ensemble Classifier**: bagged decision trees; number of learners and maximum splits automatically optimized.

**Supplemental Figure 1.** Patient enrollment

**
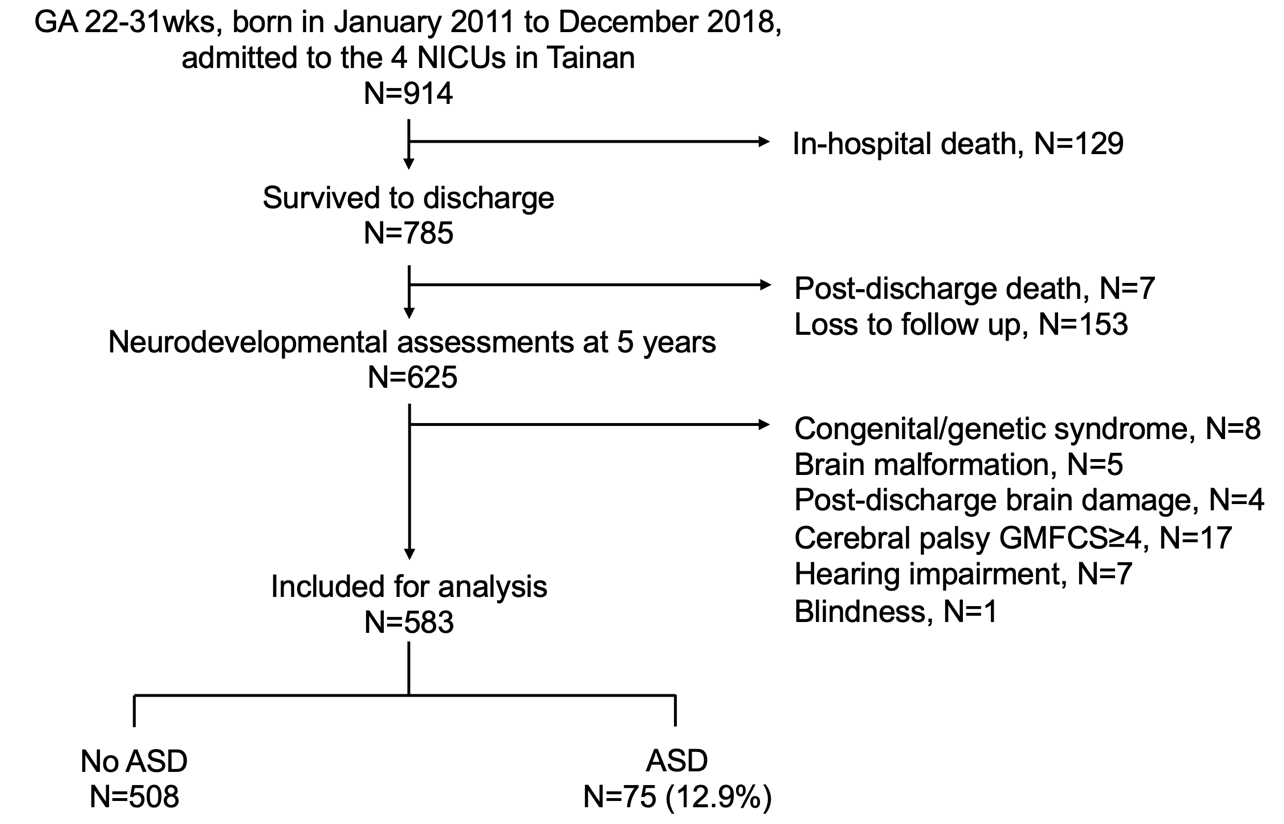
**

**Supplemental Table 1.** Demographics, neonatal risks and morbidities between children with follow-up and children lost to follow-up.

|  | **With follow-up, n=625** | **Lost to follow-up, n=153** | ***p*-value** |
| --- | --- | --- | --- |
| **Demographics** |  |  |  |
| Gestational age, weeks, median (IQR) | 28 (26.5 – 30) | 28 (27 – 30) | 0.1 |
| Birth weight, grams, median (IQR) | 1077 (865 – 1300) | 1115 (954 – 1300) | 0.3 |
| Sex, male, n (%) | 326 (52) | 78 (51) | 0.9 |
| Maternal educational level below university, n (%) | 319 (51) | 93 (61) | 0.04 |
| Low family socioeconomic status, n (%) | 184 (29) | 62 (41) | 0.01 |
| **Neonatal risks/morbidities** |  |  |  |
| Small for gestational age, n (%) | 46 (7) | 14 (9) | 0.5 |
| RDS requiring surfactant, n (%) | 246 (39) | 61 (40) | 0.9 |
| IMV duration, days, median (IQR) | 1 (0 – 7) | 1 (0 – 6) | 0.7 |
| Supplemental O2 duration, days, median (IQR) | 45 (28 – 69) | 40 (26 – 65) | 0.2 |
| PDA requiring surgical intervention, n (%) | 69 (11) | 14 (9) | 0.6 |
| NEC ≥ stage II, n (%) | 57 (9) | 13 (8) | 0.9 |
| Sepsis, n (%) | 95 (15) | 20 (13) | 0.6 |
| High-grade IVH or PVL, n (%) | 78 (12) | 13 (8) | 0.2 |
| **At discharge/Postmenstrual age 36 weeks** |  |  |  |
| Severe ROP, n (%) | 82 (13) | 23 (15) | 0.5 |
| BPD, n (%) | 199 (32) | 35 (23) | 0.03 |
| Hospital stay, days, median (IQR) | 60 (47 – 84) | 54 (45 – 79) | 0.1 |
| Body weight z-score, median (IQR) | -1.4 (-2.2 – -0.8) | -1.3 (-2.1 – -0.8) | 0.5 |
| Head circumference z-score, median (IQR) | -1.3 (-2.1 – -0.6) | -1.0 (-1.7 – -0.6) | 0.02 |

By Mann-Whitney test or Fisher’s exact test. BPD: bronchopulmonary dysplasia; IMV: invasive mechanical ventilation; IQR: interquartile range; IVH: intraventricular hemorrhage; NEC: necrotizing enterocolitis; PDA: patent ductus arteriosus; PVL: periventricular leukomalacia; RDS: respiratory distress syndrome; ROP: retinopathy of prematurity.

**Supplemental Figure 2.** Box plots of BSID-III scaled scores at 6, 12, and 24 months in the no-ASD and ASD groups. Box: median and interquartile range; whiskers: the 2.5th and 97.5th percentiles. Individual values outside this range are shown as dots.**
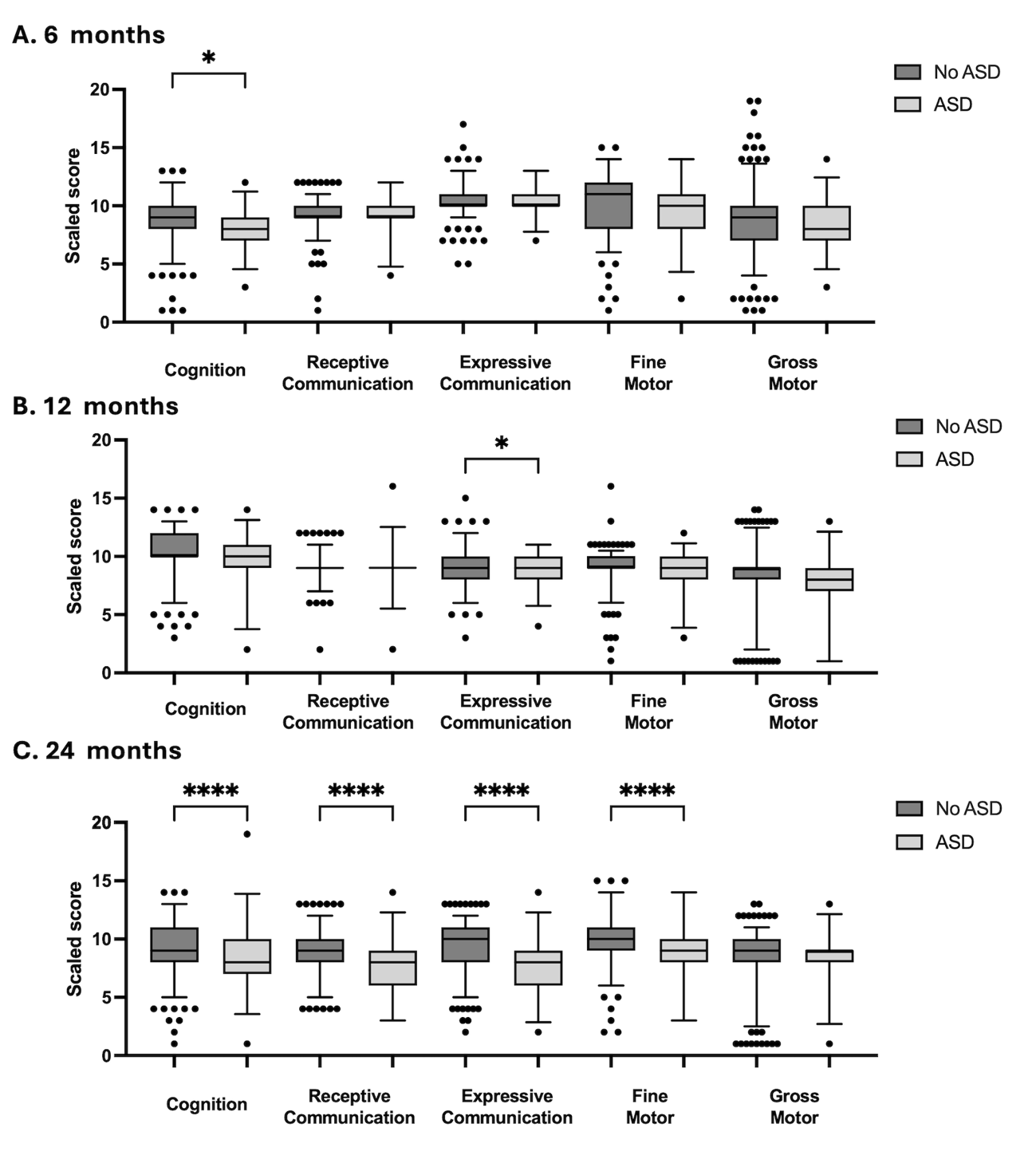
**

**Supplemental Table 2.** Model performances for prediction of autism spectrum disorder (ASD) using neonatal risks and developmental phenotypes at 6 and 12 months by the Bayley Scale of Infant and Toddler Development, Third Edition (BSID-III).

| **Feature Factors** | **Model** | **ROC-AUC** | **Accuracy (%)** | **Sensitivity (%)** | **Specificity (%)** |
| --- | --- | --- | --- | --- | --- |
| Neonatal risks plus the BSID-III scaled scores at 6 month* | Decision Tree | 0.55 | 60.4 | 59.7 | 60.5 |
|  | Support Vector Machine | 0.60 | 53.1 | 67.2 | 51.1 |
|  | Naïve Bayes Classifier | 0.59 | 64.0 | 53.7 | 65.4 |
|  | K-Nearest Neighbor Classifier | 0.59 | 52.7 | 64.2 | 51.1 |
|  | Discriminant Analysis | 0.59 | 58.9 | 56.7 | 59.2 |
|  | Ensemble Classifier | 0.58 | 56.4 | 64.2 | 55.3 |
| Neonatal risks plus the BSID-III scaled scores at 6 and 12 months** | Decision Tree | 0.54 | 54.2 | 47.8 | 55.1 |
|  | Support Vector Machine | 0.58 | 53.1 | 67.2 | 51.1 |
|  | Naïve Bayes Classifier | 0.54 | 64.7 | 37.3 | 65.4 |
|  | K-Nearest Neighbor Classifier | 0.59 | 65.3 | 47.8 | 67.7 |
|  | Discriminant Analysis | 0.59 | 60.2 | 52.2 | 61.3 |
|  | Ensemble Classifier | 0.56 | 53.8 | 58.2 | 53.2 |

*Selected features: gestational age, sex, small for gestational age, maternal educational level, and 6-month scaled scores of cognition and receptive communication. **Selected features: gestational age, sex, small for gestational age, maternal educational level, 6-month scaled scores of cognition and receptive communication, and 12-month scaled score of cognition and expressive communication.
